# Proceedings Texas State Medical Association

**Published:** 1889-05

**Authors:** 


### PROCEEDINGS

—OF THE—

### Texas State Medical Association.

### TWENTY-FIRST ANNUAL CONVENTION.

### A SYNOPSIS OF PROCEEDINGS.

#### FIRST DAY, TUESDAY, APRIL 23—MORNING SESSION.

On the bright and beautiful morning of Tuesday, April 23, there had assembled in the historic city of San Antonio some two hundred physicians, members and prospective members of the State Medical Association. Twenty-four hours later the number had doubled. They came from the north, the east, the south and the west of the great State, and represented every section and community. The occasion was the annual meeting of that body, and an unusually large number of physicians turned out, attracted, doubtless, in part, by the desire to visit the City of the Alamo, as well as to take part in the important business which this JOURNAL had announced, time and again, would come up.

Of this gathering, the San Antonio Express says: "Casino Hall was filled yesterday morning with the most intelligent body of men who ever assembled within its walls."

Casino Hall is a building admirably adapted to the purpose, having committee rooms, ante rooms, and all the requisites for holding a large convention.

The indefatigable Committee of Arrangements had everything in "apple pie order," and at 11 a. m. President J. F. Y. Paine sounded his gavel, calling to order. On the rostrum, flanking the President, sat the other officers of the Association—Vice Presidents Leake, Doak and Eastland, Secretary Daniel, Treasurer Larendon; the gentlemen of the Arrangement Committee, of which the distinguished Dr. Herff was chairman; Rev. Dr. Neal, of the First Presbyterian Church, who, being presented by

Chairman Herff, delivered an impressive prayer; Hon. Bryan Callaghan, the Mayor, and District Attorney Geo. Paschall.

After prayer, Chairman Herff delivered an address of welcome on behalf of the West Texas Medical Society, and of the physicians of San Antonio. He was followed by the Hon. Mayor Callaghan, and he by District Attorney Paschall, in appropriate addresses of welcome.

To these cordial greetings, President Paine responded in his usual happy style, when the business of the day was begun by a call of the roll. A quorum being present, roll-call was interrupted, as consuming time unnecessarily. President Paine then delivered the

ANNUAL MESSAGE

and recommendations, as follows:

*Fellows of the Texas State Medical Association :*

It gives me pleasure to congratulate you on your coming together, and hope at the outset that this will prove to be a most auspicious occasion. We have met to take stock of the year's work, to consider the progress made, and its value, to renew acquaintances and refresh friendships, and to replenish our storehouse of scientific knowledge. We have reason to be thankful to the Supreme Ruler of the universe for the many blessings we have enjoyed during the past year, and that so many of us have been spared to assemble again in the interest of professional organization, humanity and science. But some of our number are missing—have passed over to the other side, are resting from the arduous labors of professional life, and realizing, we trust, the fruits of useful and well spent lives.

It is befitting in a high degree that the healthiest and one of the most prosperous cities in the State should be the scene of the annual meeting of the State Association; and it is to be hoped that in your deliberations inspiration will be drawn from the patriotism and thrift of her citizens, to the end that a fresh impetus may be given to its onward march. Matters of great importance will come before you at this meeting, and the integrity and usefulness of this body may depend upon your action. Let me importune you to consider all matters concerning the interests of the Association carefully and conscientiously, that its existence may be perpetuated and always stand as the representative of the medical profession in the great State of Texas.

Whatever may be done in the way of remodeling the Constitution and By-Laws, conform to, and preserve the national code of ethics as the palladium of your honor.

In many sections of Europe medical men disdain the idea of medical ethics, and we are frequently reminded of the fact by foreigners who come to live among us. This feeling is readily understood when it is remembered that in those countries the governments protect the profession by rigid laws regulating the qualifications of physicians. It is difficult to comprehend the motives which actuated certain prominent doctors in some of the Northern States of this country a few years ago, in trampling upon the national code, aad openly declaring their contempt for it. They were probably imbued with European notions. However that may be, by their action they draggled in the dust the standard ot professional dignity, and degraded themselves in the eyes of American physicians. In the United States—with the exception of a few States—there are no laws protecting the medical profession from the encroachment of charlatans and impostors; the only safeguards against them are the barriers which the profession has thrown up around itself, and they should remain like a Chinese wall to all adventurers. The abolition of the code of ethics would level all distinctions between scientific medicine and the various sectarian pathies, and the shadowy and delusive systems.

The committee having in charge the revision of the Constitution and By-Laws will report at this meeting, and their recommendations should be subjected to the most searching scrutiny. There seems to be a sentiment in favor of discarding altogether the old code of laws, and substituting for it an entirely new system. I find it impossible to reconcile such a radical change with the best interests of the Association, and consequently most earnestly disadvise such a procedure. The exercises of your organization have been conducted for twenty-five years under the existing laws—such alteration and additions having been made from time to time as seemed to be necessary—and it has kept pace with the spirit of the times, and is to-day abreast with the very vanguard of similar institutions in this country.

While experience has proven that some changes or amendments of your rules are demanded, to meet the requirements of new conditions arising from rapid growth, it would be well to bear in mind the maxim : "States are best governed that are governed the least." Avoid an unnecessary multiplication of laws, for such a course will be sure to increase your troubles and impede your progress. Let all legislation be directed toward the maintenance of harmony and the promotion of scientific work. The means which would seem to be most likely to secure the accomplishment of these ends are: First, the enlargement of the functions of the judicial council so that it will have jurisdiction of all matters whatever of an unscientific nature, and the increase of its powers to the extent that its decisions shall be final as far as this association is concerned. And the scope of its authority should be defined by language so clear that no misconstruction could pervert its meaning.

Second, All business affairs should be so methodized that they could be satisfactorily disposed of without taking up the time which properly belongs to section work. Such an arrangement can be made practicable by the following provisions: Every business matter, out of the regular routine, designed to come before a session should be fully stated in writing, and forwarded at least ten days before the time of meeting to the secretary, who will keep a record of the same and report at the proper time. If useless and irrelevant discussion be barred, the morning session of the first day would afford ample time for the transaction of all business—including reports of committees worthy of consideration. A short executive session on the last day of the convention will of course be necessary. The experience afforded by the Galveston meeting has demonstrated the unsatisfactoriness of the simultaneous sitting of several sections, and the lessons learned from former assemblages, associated with the fact that the scientific work of the society is constantly increasing, would seem to demand such a revision of your order of business as will allow more time to the sections. In view of these considerations, I recommend that the morning session of the first day of annual meetings be devoted to the transaction of miscellaneous business, including reports of committees, and that the balance of the time be set apart for carrying out the legitimate objects of your organization, viz: The reading and discussion of papers. Provision must necessarily be made for the daily announcement of reports of the judicial council and a short executive session on the last day.

Recognizing the intimate relations between general medicine and state medicine, and sympathizing with the increasing interest of the profession in the latter, I respectfully suggest that article two of your constitution be so amended as to read: "The objects of this association shall be to organize the regular medical profession of the State in the most efficient manner possible; to encourage a high standard of professional qualifications and ethics; to promote professional brotherhood; to labor for the advancement of State medicine—i. e. of public hygiene (or preventive medicine), of medical education, of medical jurisprudence, and of public institutions for the sick and the infirm."

As misunderstanding and contention have grown out of the article prescribing the mode of selecting prize essays, I would advise that said article be amended, and expressed in words which will admit of no ambiguity, conferring discretionary power upon the committee on prize essays in all matters connected with the subject.

Much fruitless labor has been bestowed upon the subject of medical legislation, but we should not be discouraged. The average legislator is profoundly ignorant of the meaning of State Medicine. He labors under the delusion that quarantine embraces everything connected with the question. It cannot be hoped that a public health bill possessing the requisite force to make it effective, can be gotten through the legislature until the individual members have been educated up to a proper appreciation of its importance. In order to accomplish this end you must combine to work together. There must be no division in your ranks, no personal feelings, no local jealousies, but looking solely to the advancement of the cause, band together as brothers and no opposition will be able to resist your influence. Every fellow must be a self-constituted committeeman, and apply himself assiduously to the task of informing his representative and enlightening his neighbors upon all points relative to the subject of State Medicine. The labor connected with such an undertaking should not be estimated, because it is in the interest of the welfare of your fellow creatures—the prevention of disease and the preservation and prolongation of human life.

It would be difficult to find a citizen who could not comprehend that the state seriously needs a board of health, if its objects were properly explained to him—an institution having official supervision of the registration of vital statistics, drainage and sewerage, the structure and hygienic arrangement of building, water supply, the sale of poisons and patent medicines, food and its adulterations, the effects of trades and manufactures on the public health, the investigation of the obscure causes of contagious, infectious and epidemic diseases, measures for the arrest of contagious diseases, medical education and the licensing of physicians.

To possess the highest degree of efficacy such a board should be removed as far as possible from the domination of politics, and nowhere outside of this organization could its affairs be so advantageously administered. If precedents were necessary to substantiate this view, they are furnished by the states of Virginia, North and South Carolina and Alabama. Texas has certainly advanced far enough in civilization to consider it desirable to take measures to protect the public health, and it devolves upon this body to shape her movements in that direction.

The increasing number of suits for malpractice furnishes a subject worthy of serious thought. Incompetence does not afford a solution of all the cases, because in some instances the defendants have been among the most prominent members of the medical profession. Physicians have always been held responsible for the unfavorable termination of cases, both medical and surgical, and are frequently the victims of calumny, but their arraignment before the courts has ever been a most exceptional occurrence. The reports of trials that have come under my notice bore the appearance of having been prompted by either maiice or avarice on the one hand, or having been instigated by a doctor who had accused his associate of improperly treating a case, on the other. In each case honorable acquittal was the result, but still the accused were not nninjured, for they were not only subjected to the expense of lawyers fees and loss from neglect of business, but professonal standing was not unsoiled. It should always be borne in mind that a physician's reputation, like the character of a woman is a sacred thing, that may be irreparably damaged by a shadow of suspicion, and physicians should exercise unvarying courtesy and mutual protection in all the vicissitudes of professional life. They would not, of course, lend their influence unworthily, to shield a practitioner who, by ignorance or imposition, had gotten into trouble. There should be some local protection to society against the malevolence and rapacity of irresponsible harpies.

It is a well-known fact that physicians and surgeons are now paying a high protective tariff (45 per cent. ad valorem), on all instruments and appliances used by them in their daily routine of professional labors. A large majority of practitioners are thus prevented from properly equipping themselves, and as a consequence their patients are the sufferers. The demands of humanity cry out against this unjust duty, and an effort ought to be made to get rid of it. A number of State societies have already passed resolutions bringing the subject forcibly to the attention of the profession; and I recommend that co-operative action be taken by this body. A practical mode of proceeding would be to pass resolutions endorsing the movement already inaugurated, and instructing the secretary to enter into correspondence with all State medical societies, proffering to act in concert with them in an effort to influence congress to remove the import duty from all surgical instruments and appliances, medical supplies and medical and surgical publications.

The want of uniform quarantine laws in the different States is a menace alike to the public safety and commerce. The numerous examples of terror and alarm, and of the horrors of shotgun quarantine, furnished by the yellow fever epidemic in 1878, and more recently by the Florida outbreak; and of the losses annually inflicted on commerce by useless and onerous restrictions, clearly indicate the necessity for a more enlightened system of quarantine. The efforts of sectional interests, divided authority, and a multitude of counselors upon the administration of local quarantines, could scarcely have escaped the notice of even casual observers; and would seem to constitute a forcible argument in favor of the general government assuming charge of the entire subject of quarantine; regulating not merely the foreign, but also interstate quarantine. This object can be most advantageously promoted by the co-operation of the various State medical societies in a petition to congress setting forth the merits of the scheme.

It is the wish of my heart that the proceedings of this Association will be wise and satisfactory, that they will inure to the lasting benefit of all the fellows present; and that in your deliberations, you will not for a moment lose sight of the fact, that you are the representatives of a profession which in point of scientific attainments and usefulness is second to none on earth, and that the eyes of two millions and a half of people are upon you.

At the conclusion of this able address, Dr. E. Cross, of San Antonio, moved that the thanks of the association be tendered to the president for his interesting address, and that a committee of five be appointed to take into consideration the matters referred to in the message and to report as early as possible in regard to the excellent suggestion made. The motion was seconded and was then put to the house by the president, who, in doing so said nothing concerning the vote of thanks. It was carried, when Dr. J. H. Sears, of Waco, drew attention to the omission, eliciting a response from the chairman that he thought officers scarcely deserved thanks for doing their daty. The chair appointed on the committee Dr. E. Cross, Dr. H. K. Lake, of

Dallas, Dr. Sam R. Borroughs, of Raymond, Dr. A. V. Doak, of Taylor, and Dr. L. J. Graham, of Henderson.

The roll of the judicial council was called, and only Dr. M. D. Knox, of Hillsboro, Dr. Isaac E. Clark of Schulenburg and Dr. T. J. Bell, of Tyler, answering to their names, the president filled the vacancies temporarily as follows: Dr. G. W. Kerr, of Waelder; Dr. J. V. Spring, oi San Antonio; Dr. W. C. Fisher, of Galveston ; Dr. J. M. Pace, of Dallas; Dr. E. W. Rush, of Paris; Dr. T. H. Nott, of Goliad; Dr. J. H. Sears; of Waco; Dr. L. J. Graham, of Henderson, and Dr. W. P. Powell, of Willis. Dr. W. L. York reported later and took his place on the council.

The council organized by electing Dr. Knox president and Dr. Bell secretary.. They retired to the ante-room and the secretary brought up the report of the committee on constitution and by-laws, of which Dr. Burroughs acting chairman. Dr. Borroughs said that if the convention were to begin on the report then it would probably last a week, and if they considered it section by section they would hardly ever end. He therefore moved that the consideration of the report be postponed until 10 o'clock Friday and that it be made the special order of the day. This motion was seconded and finally carried.

Dr. E. Cross moved for a reconsideration of the motion as he thought it would be well to consider it before that, because on that day an excursion over the Aransas Pass Railroad had been arranged. The motion to reconsider received a second but the chair ruled it to be out of order. He could not entertain any motion that would subordinate the business of the association to purposes of pleasure.

Dr. Cross said he thought the motion was right and he would have to appeal to the house in that matter.

The chair repiied that the motion was badly taken because it was moved for a definite purpose and had no connection with the objects of the meeting, and thereupon Dr. Crew withdrew his motion to reconsider.

The special committee of Ophthalmologists (Dr. G. P. Hall, chairman,) appointed at Galveston to examine the paper ("Opthalmoscope in General Practice") and which had been selected by the Prize Essay Committee "for presentation to the association" and recommended for the $100 prize, was called on for their report. No member of this committee being present, no report was forthcoming. Later a report was received, but the matter having been disposed of by Dr. Nott's resolution, as will be seen presently, the report was suppressed.

Dr. T. H. Nott, of Goliad, said that there was ambiguity in the article of the constitution referring to it, and moved that the motion creating a second committee on prize essay be reconsidered. The motion to reconsider was seconded and adopted.

The mover of the resolution then moved that $100 be paid as reeommended by the original committee.

Dr. F. E. Daniel, of Austin, said that the condition precedent to the award was that the paper should have been read to the association in open session and approved and ordered printed, and the award would not then be in direct violation to the constitution and by-laws. He protested against any hasty action being taken which would lay them open to the charge of proceeding in violation of the constitution and by-laws. The association had never seen the paper—it had not been read and he asked that the matter be brought before the association in the status that it occupied before it came before the special committee.*

Dr. Cupples said that the question presented appeared to him to be a very simple one. The power delegated to the committee left no discretion to the association and they were not setting in jndgment on their action at the time. The question was delegated to the committee and their action was a finality.

The question was then put and carried, Dr. Daniel voting in the negative.

Dr. A. V. Doak, of Taylor, chairman of the original Committee on Prize Essays, arose to a question of privilege, explaining his position and showing how mortified and chagrined the committee had been at the action of the Convention, and that by the present action they felt that they had been justified, and he thanked the Convention for vindicating the committee.

[*No action was had on the paper itself; it was not "received," nor "referred to the publishing committee," hence it was returned to the author.—Ed.]

The report of the Committee to Memorialize the Legislature to set apart a room in the capitol for the use of the Texas State Medical Association was called up. The Secretary announced that the chairman, Dr. F. R. Martin, of Kyle, was absent, and therefore there was no report, but he informed the Association that he knew the Legislature had refused the petition.

Dr. H. K. Leake, of Dallas, again brought up the prize essay question, and said the prize essays had been a source of unwholesome agitation, and he moved that the matter be abolished entirely from the proceedings and that the section providing for the appointment of any committees on prize essays be wholly abolished.

Dr. Cupples seconded the motion for the reason that it would be a source of heart burnings and dissensions.

Dr. H. C. Ghent, of Belton, supported Dr. Cupples' position, and the question being put to the Convention, Dr. Leake's motion carried, but a small minority of negative voices being heard.

The report of the Committee on Legislation was called for, when Dr. Couples, the chairman of the committee, asked that a special order be made for the report, as it was a long one and embraced questions of vital importance to the people of the State and the profession, and required some consideration on the part of the Convention.

Dr. Daniel seconded the motion, and it was carried, the report being made the special order for Wednesday at 10 o'clock.

The report of the Committee on Surgical Cases was next asked for, of which Dr. Cupples was chairman. Dr. Cupples announced that no report on surgery had peen prepared for that session, other labor in behalf of the Association rendering it impossible for him to complete the requirements of the task, and he hoped he would be allowed to present a biennial report. The time asked for was, on motion, granted.

The report of the Secretary was read, and on motion of Dr. Leak it was received and referred to an Auditing Committee, consisting of Drs. Leak, Powell and Graham.

The report of the Treasurer, Dr. J. Larendon, of Houston, was also read, and referred to the above committee. Also report of Publishing Committee.

The Secretary tendered the resignation of Dr. Florence E. Collins, of Austin, as a member of the Association, which was accepted.

The Secretary announced that an exhibit of surgical instruments and a pharmaceutical display were being held in the new dining room of the Menger hotel.

The secretary read a communication to this society and other State medical associations asking co-operation on revision of the pharmacopeia of the United States of America.

Dr. A. N. Denton moved that a committee be appointed to take charge of the communication and report on it. The motion was seconded and carried and Drs. Denton, Daniel and Smith were appointed as a committee by the president for that purpose.

Dr. Thruston, of Dallas, called the attention of the association that there were several visiting members of the profession and moved that they be invited to seats on the floor as the guests of the association. The motion was carried and in accordance with it Drs. J. F. Noyes, of Michigan; Grant, of Cincinnati, Alvis of Arkansas, and Cain, of Nashville, and Stewart, of Mississippi, were invited to take seats.

The president then announced that as the business of the day had been disposed of it would be in order for the sections to begin their work.

At this juncture a motion to adjourn until 3 o'clock was made and carried. The convention then adjourned.

### FIRST DAY.—AFTERNOON SESSION.

The president called the convention to order shortly after 3 o'clock and announced that the business of the afternoon would be that of work in sections.

### THE SECTION OF PRACTICE OF MEDICINE, MATERIA MEDICA AND PATHOLOGY

was called up and in the absence of the chairman, Dr. R. B.

White, of Ennis, Dr. J. C. Loggins, of Ennis, was appointed chairman by the president and Dr. Rosser, secretary.

The first paper read was an able and exhaustive one on

CONTINUED FEVERS IN TEXAS, by Dr. H. A. West, of Galveston, in which he argued that there was no typhoid fever in Texas. A very animated and long discussion followed, publication of which is precluded by pressure on space. Dr. Paine, Dr. Fountain, Dr. Thruston, Dr. Sears, Dr. Pettus, Dr. Knott, Dr. E. J. Ward, Dr. Cross, Dr. Dodd, Dr. Dock, Dr. Leake, Dr. A. W. Fly, Dr. M. A. Taylor and Dr. J. B. King, all took an active part in the discussion and the consensus of opinion was antagonistic to the opinions advanced by Dr. West. On motion of Dr. M. A. Taylor, of Austin, the paper was received and referred to the publishing committee.

Dr. A. M. Autrey reported a case of *enlargement* of *the thyroid gland*, which was received and referred to the publishing committee, after which the section adjourned until 9:30 next morning.

SECOND DAY, WEDNESDAY, APRIL 24TH—MORNING SESSION.

The President called the meeting to order at 10 o'clock. Prayer by Rev. Mr. Scudder. Minutes of preceding day read and approved.

The Committee on Legislation was called. Dr. Geo. Cupples, chairman of the committee, then submitted the report, prefacing it by saying: "Your committee on legislation has had under consideration a subject of utmost importance to the people of the State, questions that come home to every heart and affect the welfare of every member of the community. The obstacles that have been met with all of you have some knowledge of but only those who have been engaged in trying to petition the legislature on this subject can form an adequate idea of the difficulties we have had to encounter.

The report of your committee is as follows:

### REPORT OF THE COMMITTEE ON LEGISLATION.

*J. F. Y. Paine, M. D., President of the State Medical Association:*

Your committee on legislation have the honor to present their report as follows:

It seems to your committee that two principal measures are imperatively demanded by the necessities of the people and by public policy rightly understood. These are, first a law to restrict the practice of medicine and surgery in such manner that henceforth, in this State, no one shall be permitted to practice the healing art until his qualifications for that office shall have been ascertained and recognized in a manner prescribed by statute.

We would premise that no law on this subject could have any retroactive effect and that it is distinctly admitted and understood that all legally qualified practitioners who have become such by prescription through length of practice, or by compliance with any law now or heretofore in force, will retain their status on registering in the manner provided in the draft of a bill to regulate the practice of medicine which accompanies this report.

Before entering on the consideration of this very difficult and important matter, we propose to relate in a succinct manner the history of the legislation which has been had on this subject, as also the means and modes of procedure which have in communities of longer experience than ours been found most available in surmounting the difficulties inherent to the subject.

Thirty-six years ago, in 1853, the first effort to procure the anactment of a law regulating the practice was made by the State Medical Assoeiation, and was continued at intervals without result until 1873, when a bill was passed requiring the registration of diplomas by new comers to the State. Another law was passed in 1876 repealing its predecessor, on which it was a great improvement, inasmuch as it created boards of medical examiners, whose duty it was made to examine all applicants for license, whether graduates or not. This law though available in many of its features, contained defects which made it a failure. By a subsequent amendment in 1876 it was completely emasculated and county clerks were required to record all diplomas from recognized medical colleges, which might be presented for that purpose. This record constitutes the license.

Let me ask, gentlemen, if any county clerk in our State is competent to say what medical colleges are recognized; nay, more, is there a county clerk in the State who could intelligently read the diploma presented to him for record? This was a burlesque on legislation which surpasses all comprehension.

Recurring to the statutes of 1876, the only one of these enactments worthy of consideration, it contained one fatal defect in that it failed to define the offense; and the article in the penal code relating to it remained inoperative in consequence of this omission.

Again, the number of examining boards was too great, there being one for each judicial district; and this, in itself, precludes any approach to uniformity in the standard of qualifications required of candidates, and rendered impossible any authentic record of the proceeding of the different boards, from which an official register of the licensed practitioners in the State might be prepared.

"Another well founded objection was to the source of appointment, this being the judge of the district court, who, however learned in the law, honest in intention, upright as a man and able as a legal functionary, is hardly a competent judge of the qualifications of doctors, who, in their turn, are to sit in judgment on the competency of others. I could cite instances of ludicrous blunders made by able and honorable district judges in the appointment of medical examiners."

To remedy these evils and to prevent others, it is manifest to every one that we must begin *de novo;* that we must prepare a draft of a bill which shall embody the following features:

First: A comprehensive and incontrovertible definition of what shall constitute the offense of practicing medicine or surgery without proper authorization.

Second: The appointment by the Governor of the members of the Board of Medical Examiners for the State of Texas, who shall devise an equitable mode for examination, which will insure, as nearly as possible, a uniform standard of qualification on the part of the applicants for license.

Third: An official register of all the qualified practitioners of medicine of the State under all the laws regulating the subject matter, which register shall be an official record, and prima facia evidence of what it contains.

Such a measure entirely ignores a diploma as a license to practice medicine, and will effectually prevent the personation of graduates by persons who may have become possessed of their credentials by fraud.

This feature may strike many of you as a daring and dangerour innovation; but in truth it is not so, but it is a fundamental principle in the legislation governing the practice of medicine in European states.

In the German Empire the possession of a diploma conveys no right to practice the healing art; the graduate must pass an examination before examiners appointed by the State, and receive a license from them before he can enter on practice. In France all educational institutions, public or private, are under the control and supervision of the Minister of Public Instruction and of the University of France, who appoints all examiners in every department of knowledge, who are not teachers; consequently in France a diploma in medicine constitutes a license, as having been conferred by authority of the State. In Great Britain degrees are granted by the University after examination by the *Senatus Academicus*, and diplomas by the different licensing bodies, such as the Royal Colleges of Physicians of London and Edinburgh, and of Surgeons of England; of Ireland, of Edinburg, Faculty of Physicians and Surgeons of Glasgow, etc., etc., but assessors are present at all examinations for a medical degree or license. These assessors are appointed by the general council of Medical Education and Registration, who have supervision of all matters in relation thereto, in virtue of their authority derived from the State, so that the degrees are licenses from the State itself.

The University of London, the degree from which is so highly prized, is not a teaching body at all, but recognizes as a title to it the possession of the requisite acquirements, however and whenever they may have been obtained. The principal is being more favorably discussed every day, and measures are being agitated in accordance therewith.

Many States of the Union have adopted it, and the statutes regulating the practice of medicine are based upon it. The supreme courts of Illinois, West Virginia, Massachusetts, North and South Carolina, Missouri and Minnesota and others have unanimously, we believe, affirmed the right of board of examiners to refuse to recognize the diploma as a license to practice. They have affirmed the constitutional right of the State, in the exercise of its power of general police, to enact such laws as they may deem necessary for the protection of their people in this relation.

We now arrive at the radical evil, the *fons et origi mali*, recognized as such by all who have treated this subject—and that is the multiplication of schools of medicine in the different States through the blamable facility with which charters are granted for that purpose, no more formality or guarantee being required than is necessary to secure a charter for a company proposing to manufacture any commodity for daily consumption.

There are schools of medicine in this country whose diplomas are an honor to their holders, yet even they, by the common consent of the profession, are acknowledged to be defective in some respects, and more especially in the preliminary and literary educaiion of their matriculants. Much improvement has taken place in these respects of late years, and more is to be desired.

Yet, what shall be said of the innumerable schools created by State charters, and unequal to the task of medical education, through want of facilities, means and appliances indispensably necessary to that end, however great may be the ability of the professors, an element of success not always present?

The natural and inevitable result is, that crowds of graduates have been let loose on the people, possessing a mere smattering of medical knowledge, acquired in some of these institutions in fifteen months; while in the great schools of the old world, from four to seven years are thought to be none too much for the purely medical education of well-trained, and well prepared students.

Again, a charter with power to grant degrees in medicine, can be of force only in the State granting the charter, and it can in no way be regarded as a breach of equity, or a violation of comity, if their neighbors decline to recognize doctors of their manufacture.

These considerations, mainly brought forward in the discussion throughout the United States of late years, have led to legislation in the majority of them, and the urgently felt need of uniformity of qualifications has led to the introduction into the Senate of the United States of a bill looking to the creation of a central authority to examine and license practitioners of medicine for the entire union. This measure, politically, perhaps of doubtful expediency, would, from a professional standpoint, solve all the difficulties of this much vexed question, and inure to the benefit of the vast population whose dearest interests it concerns.

Some State boards have exercised the right to discriminate between the sufficient and insufficient schools of medicine, recognizing the diplomas of the one class, and rejecting those of the other. We have deemed it best and safest to adopt the principle and rule recognized by the highest authorities, and to admit but one test, that of examination; thus ignoring diplomas, in view of the difficult and doubtful task of discrimination.

The failure of our legislature to enact an efficient law to exclude incompetent practitioners, has resulted in a most disastrous condition of matters medical; the people having no means of distinguishing between qualified and unqualified practitioners of medicine.

Charlatanism and imposture, the offspring of ignorance reign rampant in the land; no legislative check restrains the indiscriminate and unregulated practice of physic by unqualified persons, the incredible and destructive abuse of nostrums and secret remedies; humbug is the order of the day.

The enactment of stringent laws to regulate the practice of medicine in most of the States of the Union, in which course Illinois led the way, guided by that eminent reformer, John H. Rauch, Secretary of the Illinois Board of Health (oh, for a Rauch in Texas!) has driven hordes of quacks of all known and unknown varieties from their accustomed haunts to seek more genial climes, and our fair land of Texas has been an asylum and a a refuge, and they have come in swarms to prey upon our people.

Of all grades, from the magnificent Bostonian who travels in his special car heralded by an obsequious because well paid press, to the humble "yarb doctor," who culls his own simples, and the medical magician who diagnoses the case and effects an infallible cure after examination of a lock of hair of the helpless patient; and the cry is still they come.

To remedy this crying evil repeated and earnest efforts have been made by the association and individual members of the legislature, only to meet with reiterated and disastrous defeat, although laws to regulate the important yet secondary branches of practice, to-wit: dentistry and pharmacy, have been enacted. The apathy, the incredible indifference of successive legislatures on this point merely proves that they have not yet learned the first duty of a government which is to protect the lives and health of its people.

One word as to the part taken in this movement for reform by the physicians of Texas. They have been charged with selfish motives as underlying their action in this matter. This charge defeats itself, and the puny enemies of reform, it would seem, are satisfled with the present condition of the law, and claim to believe that the people are sufficientiy protected in their lives and health. The 4,000 physicians of Texas, in their twofold capacity of citizens and guardians of the public health, are, with few exceptions, of a different opinion, and knowing their duty, dare to perform it. Those who object, in their failure to sustain this movement, have no doubt potent reasons, which we will not seek to investigate. We are called on to prepare legislation for Texas, not as she is to-day, but for the time when she shall count her sons by tens of millions—when she shall be in population as in grandeur and extent, in power as in resources, the peerless State of the Union. The convention which framed the constitution of 1845 proved themselves true statesmen when they by the organic law, provided for the cultivation and mental health of the children of Texas the unparalleled scholastic fund which to-day amounts to $90,000,000, and a grand University has arisen in our midst, which if the present administration be equal to their task will leave Texas nothing to envy in the institutions of learning in the great centers of old. A magnificent building has been erected in which her legistors hold their deliberations, and which is the symbol and the monument of Texas patriotism and State pride, surpassing as it does in grandeur and extent every work of human hands on this hemisphere saving only the National Capitol. Thus have we munificently provided for the intellectual and politieal wants of our people; but for the protection of their bodily health, without which all other blessings are but a mockery, not a dollar has been expended, and a mere burlesque of a law has been enacted.

The bill now presented* for your consideration is based on one passed by the General Assembly of Ohio, which was the outcome of the experience of the working of laws to the same end in other States, and in European countries, and which has been modified, and, as we believe, more nearly meets the needs of our people, and the aim proposed, than any law that has been proposed to any State Legislature. It is the result of much thought and most careful consideration, after collation and comparison of analogous statutes in other States, and of the report of the working of these laws. It will be useful to analyse, very shortly, the salient features of the bill.

The principal features of the bill under discussion, are as follows:

1. It violates no constitutional provision.

The State Constitution contains a clause to the effect that no discrimination shall ever be made in favor of any one school or system of medicine. It is true that this bill was drafted under instructions from the State Medical Association, representing the regular or Hippocratic school of medicine, of which there are over 4000 representatives in Texas, while those of the Homœopathic and Eclectic schools uunber less than fifty, yet we defy the most astute, the most acute lawyer to detect in this bill any evidence of its origin. There is no allusion to any difference of schools, any diversity of opinion.

It provides that a fair and impartial examination shall be made of all candidates for license, in anatomy, physiology,

---

*The draft of the bill did not accompany the report, and has not reached the Secretary's hands.—Ed.

pathology, hygiene, surgery, chemistry and obstetrics, all which departments of science are recognized by all shools of medicine, and knowledge of them required by such, as the basis of all medical knowledge. Practice of medicine, on which alone the schools differ, is not included, nor is materia medica or therapeutics.

2. It has no retro-active effect; all legally qualified practitioners who have become such by prescription through length of practice, or by compliance with any law now or heretofore in force, retaining their status.

3. A single board of examiners appointed by the Governor.

4. An official register, which will be an authentic record and official proof of the professional status of those whose names shall be borne upon it.

An objection may arise to the creation of a single board of examiners for so vast a territory.

It is a self-evident truth that when responsibility and authority are exercised through many different agencies, it becomes inefficient.

Again, the want of uniformity in carrying out the objects of their creation constitutes a serious argument against the creation of several boards.

The system of having a board for each judicial district must evidently be abandoned.

The only territorial circumscriptions available for the purposes of this measure would be the congressional districts, eleven in number. A glance at a map of the State will soon satisfactorily show that all the districts are within easy access of Austin, with the exception of the Eleventh, or Shoestring District, in which El Paso, Laredo, Corpus Christi, Brownsville and Galveston are situated. These places are accessible to each other only by passing through or within eighty miles of the State capital, which this bill makes the seat of the board.

Furthermore, by the formation of a board from the State at large, all local influences and prejudice, for or against candidates, would be effectually obviated.

The importance of the medical register cannot, in our opinion, be over estimated. Proffessor Stanford E. Chaille, of New Orleans, in a very able paper on State Medicine and State Medical Societies, to be found in the Transactions of the American Medical Association, speaks thus:

"The only law ever yet enacted on this subject by an English-speaking people, which, tested by experience, has proved worthy of imitation, is the British Registration law of 1858. This law is reported to have effected great good. It is executed by a General Council of Medical Education and Registration, and it is based on the principle that "it is expedient that persons requiring medical aid should be enabled to distinguish qualified from unqualified practitioners.. Those whom we designate irregulars are, if found qualified, registered equally with regulars, and fraudulent registration is liable to severe penalties. Only those physicians who are registered are recognized by the courts, are exempt from jury and militia duty, and enjoy the rights to practice and collect fees, to sign legal certificates and to serve as medical officers in the army, navy and civil services."

This Official Register has been incorporated in the laws regulating the practice of medicine in several states, to great advantage, notably in Missiouri and Louisiana.

REMEDY FOR EVILS.

And now you will ask, what is proposed as a remedy for this deplorable condition of matters medical in our state?

Your committee unanimously recognize the futility of the most earnest endeavors to arouse the legislature to a sense of their duty in this regard, and now have the honor to propose a combined plan of action by the profession and the people, suggested by one of their number several years ago, having as its chief features:

1. Education of public opinion by the medical profession throughout the state by constant discussion in season and out of season.

2. Making the practice of medicine bill and board of health enactment an issue in the next election of state senators and representatives.

3. A monster petition from the people throughout the state, urging these measures.

The organization of this contemplated campaign by the committee on legislation, which will suggest a uniform and concerted plan of operations, comprising the furnishing of necessary documents to a committee of physicians in each county in the rural districts, to the local medical societies and to the physicians in towns in such manner as to unite and systemize their efforts to this great end. Let the physicians of Texas, hand in hand and shoulder to shoulder, inaugurate a crusade against legislative supineness and professional abuses; let them arouse the people by explaining the situation, and who can doubt the result? Shall this great state, with its well-founded, if lofty pretensions, prove a laggard in the career of civilization, and allow the reproach to continue, "that human life is the cheapest of commodities in Texas?"

Respectfully submitted,

GEO. CUPPLES,
Chairman Committee on Legislation.

In conclusion, Dr. Cupples advocated the formation of a State Board of Health, having charge of all matters sanitary. The central board of health, he considered, should collect and utilize the vital statistics furnished by local boards of health. In regard to the operation of local boards, the first thing was for them to prove that a man was born, but there was no such law in Texas; then the board should prove when a man dies, but at present there was no authentic proof, unless by oral testimony, which, after one generation disappeared. There was a law on the statute books that every death should be recorded by the county clerk, but it was not done. It was also absolutely necessary, he considered, that a quarantine officer should be established independent of any deliberative body; he should be a member of the personal staff of the governor and that he should be able to use discretionary, and at times, arbitrary power, and not be subject to the directions of a legislative body.

The report was received with much applause, and on motion of Dr. Loggins the report was unanimously adopted.

Secretary Daniel moved that the report be referred to the publishing committee with instruction to print, and that a vote of thanks be tendered to Dr. Cupples for his able address. This motion was seconded and carried.

The secretary then moved that in view of the great comprehensiveness and completeness and ability ot the address, the secretary be instructed to have 5,000 copies printed for distribution throughout the State. This motion was seconded.

The chair said that the financial condition of the Association was none of the best, and he thought it would be well to provide for the publication of the report by some other means—by a special tax, or by the prompt payment of back dues by members.

The secretary drew attention to the fact that it could be done in the way of reprints from the Transactions, if there was money enough in the treasury to pay for it.

Dr. J. H. Sears amended the motion by moving that the committee be continued and that the publishing committee be instructed to send out the report to every physician in the State in addition to a blank petition to be put before the people for signature and to get them ready by the next legislative meeting. He thought the State ought to have power to supervise all licenses and believed they were the only profession that were permitted to take any kind of license from any kind of authority and go forth and practice medicine.

Dr. Denton asked the secretary if he could form an approximate estimate of printing.

The secretary replied he was unable to give an exact estimate, as it would depend on the number of pages, but that about $1.00 per page per thousand would cover it.

Dr. Denton said his object in asking was that he was in favor of having 10,000 copies printed instead of 5,000.

Dr. R. M. Swearingen said that while he was in perfect sympathy with the paper, he must oppose the proposition to distribute 5,000 copies to the people of Texas. They might as well distribute 5,000 to the Gulf of Mexico, for what good would it accomplish to the people of Texas! They had 200,000 or 300,000 voters, and 5,000 would not change the opinion of one single man in Texas as to the necessities of legislation. They must move in slow but progressive steps in that line of enactments. The members of the Legislature were not going to accept anything that looked like dictation from medical men. But by slow degrees in their homes let them talk to the people there, and they were then going to march steadily on to the triumph of the suggestions contained in the paper. In fact, there were indications already that light was beginning to come to the people of Texas. The drug bill he would mention that had passed and without opposition, and in a short time the druggists of Texas would have to go before a board and pass an examination before they can mix up prescriptions. That showed that daylight was breaking in the east, and the progress they were making. He wished to say he was in perfect sympathy with the paper and all its objects, but it was no use to waste the money; they were not millionaires, in fact, their bank ran rather low. He proposed to oppose the resolution, and would offer to table it were it not for discourtesy.

The Secretary said he was forcibly impressed with the truth of Dr. Swearingen's remarks, and in consideration thereof begged to withdraw his motion.

Drs. Robertson and Taylor discussed the subject, favoring the publication and distribution of the report.

The chair said that there was an amendment before the house to print 5,000 copies of the report.

Dr. Denton said that the amendment had been withdrawn.

The chair said the seconder of the amendment had not consented to its withdrawal.

Dr. Autry said he had seconded the motion of Dr. Daniel but would withdraw it.

The chair then announced that the motion before the house was a motion to continue the committee and publish 5,000 copies, two copies to be sent to each of the county commissioners' courts in the State.

Dr. J. Cummings moved that the committee postpone its final report until the next meeting.

Dr. J. B. Robertson seconded the motion and this motion being put was carried.

The chair announced that the committee after reading the report were to be considered discharged, when Dr. Osborn moved that the committee be continued. The motion was seconded and carried.

At this juncture the judicial council made their report as follows:

The judicial council beg leave to make the following report:

Applications of the following gentlemen were received and approved: [See list elsewhere.]

Respectfully submitted,

T. J. BELL, Sec'y.

The report of the judicial council was received and adopted.

The Secretary announced receipt of the report of the committee of ophthalmologists appointed at Galveston to report on a prize essay and asked what disposition should be made of it.

The chair said that it would come up under the head of unfinished business.*

The President then resigned the chair to Dr. J. C. Loggins, the convention entering upon section work. The

SECTION ON PRACTICE OF MEDICINE

being in order, Dr. Dock gave an interesting demonstration of malarial parasites.

On motion of Dr. E. J. Ward, Dr. Dock was asked to put his remarks in writing and it was carried.

Dr. West, of Galveston, endorsed Dr. Ward's motion and highly complimented Dr. Dock.

Dr. Dock then read a paper on leprosy, giving a report of two cases which had come under his notice at Galveston. Prior to reading the paper Dr. Dock circulated among the members two large photographs of the subjects that had come to his notice. The discovery of such a disease, said Dr. Dock, arising apparently *de novo* in any place, would naturally lead them to inquire whether there exist any peculiar climatic telluric or dietitic influences, such as have been looked on in other times and places as being effective. They saw in Galveston, as in many endemic localities of leprosy, a low-lying sea-coast situation, but they could not from their knowledge of the disease in other parts of the world see any definite predisposing cause. So far as the effects of diet or of mode of living were concerned, it was to be noted that both cases reported were in the class of people who were most highly favored in their material surroundings, leading lives devoid alike of want and excess. With regard to heredity, it could be positively excluded in both cases, as could also an importation from the old world, by the patients themselves. The inference that was compelled to be drawn, therefore, was that both patients acquired their leprosy in Galveston and in a manner so far unknown. In these cases there was a lesson to be

*[It never came up, having been superseded by Dr. Nott's motion.—Ed.]

learned regarding the contagiousness of the disease. They were in these cases at once met by the vague signification of the term contagion. The two patients, for a period aggregating at least thirteen years, have been moving among their fellows, living in the most intimate relations with wives and children, yet so far no other case of disease has been acquired from them. Dr. Dock then recited a history of the two cases and a brief biographical sketch of the two men, describing their appearance and present leprous condition.

The reading of the paper was listened to with great attention and at its conclusion Dr. Noyes, of Detroit, gave his experience in Honolulu, where he visited the leper hospital. While there he inquired about the contagiousness of the disease and the physician informed him that leprosy was not contagious in the sense generally understood by contagion, and that it does not spread in families who were living close together. He only saw one white person in the hospital and it is remarked that the disease does not seem to have made any headway among the whites. It seemed to be found among the natives altogether.

Dr. Denton moved that a vote of thanks be passed to Dr. Dock and that the paper be referred to the publishing committee. Dr. Ghent seconded the motion and it was carried.

Dr. Osborne, of Cleburne, next read a paper on

RŒTHELN, and pointed out its dissimilarity to scarlet fever, and after the paper was read it was referred to the publishing committee.

Dr. T. H. Nott said he wanted to hear some gentleman say something on malignant "Rœtheln."

A general discussion concerning the disease and its similarity to scarlet fever then ensued; Drs. T. H. Nott, Leak, Denton, West, Ghent, Autrey, D. M. Ray, Fontaine, F. T. Paine, Evans and Lancaster, of Moulton, taking part.

There being no other business before this section President Paine again took his seat and called up the

SECTION ON SURGERY AND ANATOMY, appointing Dr. Swearingen, of Austin, as chairman in the absence of Dr. J. C. Jones, of Gonzales, the regular chairman.

Dr. Swearingen called the section to order and appointed Dr. J. D. Osborne as secretary.

Before proceeding with the business of the section, President Paine asked the convention concerning the status of papers read and referred to the publishing committee. The question was, whether after being read, the papers were to be considered the property of the association, and members were bebarred from publishing them in any periodical prior to the publication of the regular transactions?

Dr. Sears said he did not think they had a right to publish them unless they had the consent of the association, and he made a motion to this effect which was seconded and carried.

Dr. E. J. Ward, of Waxahachie, then read an eleborate and interesting paper on

PREVENTIVE SURGERY, in the course of which, for the purpose of elucidating his subject, he touched on the questions of evolution and heredity. The paper attracted general attention and it was referred to the publishing committee.

It being at this time nearly half past 1 o'clock, on motion of Dr. Denton the section adjourned until 3 o'clock, discussion of the paper being postponed until then.

AFTERNOON SESSION.

The convention was called to order at 3 o'clock; and, in accordance with the adjournment, the Section of Surgery was reopened and discussion of Dr. Ward's paper begun.

Dr. Denton said, he did not rise to discuss the paper but he desired to commend it as an admirable one on the subject.

Dr. Sears said, he must compliment Dr. Ward for the style of his paper; it was gotten np in a very good style and he admired it very much so far as the style was concerned, but not a word of it did he believe to be true. He claimed that his progenitors came from a man in every respect who always stood erect, and that he was never more degraded than man was to-day. One who always walked erect, had two legs always and hands, and the same developement of brain he had to-day, and probably in a larger measure, because he thought the first man more perfect than any since. He believed the generation of to-day sprung from Adam and that they never came from any member or anything inferior to himself, and so far as development was concerned, that he developed from a man down, if anything. Dr. Ward combated Dr. Sears' assertions, and after considerable argument the discussion closed.

The next paper was read by Dr. Q. C. Smith, of Austin on

CHLOROFORM AS AN ANÆSTHETIC AND HOW TO USE IT.

This paper was received and referred to the publishing committee and extensively discussed. Dr. Ghent rose to heartily endorse the paper, and various members gave their experience with chloroform and ether as anæsthetics, among them being Drs. Denton, Fontaine, Clark of Schulenburg, Doak, Oliver, Polk, Rosser, Paine, Noyes, Herff and Spohn. The majority of the speakers inclined more favorably to chloroform than ether as an anæsthetic. Dr. Herff speaking emphatically in its favor after having used it thirty-eight years.

The next paper was read by Dr. J. P. Oliver, of Caldwell. It was on

PERITYPHLITIS, and was referred to the publishing committee without discussion.

Dr. Bennett, of Austin, then read a paper on

ANAL FISTULA WITHOUT CUTTING OPERATION.

This was discussed by Drs. Carhart, Lancaster, Doak and Fontaine, and after the last speaker Dr. West, of Galveston, drew the attention of the section to the hour; it was 5 o'clock, and on motion the section adjourned until Thursday morning at 9 o'clock.

The president again took the chair and announced that the committee of arrangement had invited the members to take a ride around the city at 5:30 and hacks were waiting for them at the Menger hotel.

Dr. F. Herff stated that all members desirous of going on the Aransas Pass excursion to Corpus Christi should register their names separately in order that the railroad company could provide necessary transportation. He announced that the train would start on Friday afternoon at 2:30 and would return from Corpus Christi on the following day at the same hour.

The president asked that the nominating committee report in the morning the first thing, and then on motion the convention adjourned until 9 o'clock Thursday morning.

THIRD DAY, THURSDAY, APRIL 25.—MORNING SESSION.

The meeting was called to order at 9 o'clock, President Paine in the chair. Prayer by Rev. Chas. E. Giddings. The minutes of the previous day's session were read, amended and adopted.

The Nominating Committee was organized, and retired for work.

The action had yesterday, postponing the publication of the report from Committee on Legislation was brought up by Dr. E. J. Ward, who, referring to the press reports of the day, said he was in the Judicial Council room yesterday when this matter came up, and until he read in the Express the elaborate report, he had no idea of the stupendous amount of work which it represents, and thought it due to Dr. Cupples, whose work for the Association has always been regarded as the best that has been done, and he has never done anything abler than this report, that the report should be published, not only in the Transactions but for circulation, and he moved therefore to reconsider the vote postponing action.

Seconded by Dr. West.

Dr. Daniel said that Dr. Cupples felt that his labor had been in vain, if no use is to be made of it, and that he has been placed in a false light before the public; that the report of the action had, conveys the idea that it had not been approved or appreciated by the Association; in fact, that he had been "sat down upon," and that although he, Dr. D., had explained to him that no such thing was intended—that it was only a question of financial ability to publish and distribute the report—still the doctor felt hurt. Dr. Cupples' idea, he said, was not to distribute the report promiscuously, but to work through a committee of physicians in each district.

Dr. Cummings, the mover of yesterday's resolution to postpone, explained that no discourtesy or want of appreciation was meant; simply that he thought to send out the report now would be premature, the proper time being, in his estimation, just before the next legislature meets.

Dr. Ward's motion to reconsider prevailed, and after several motions and amendments looking to the number of copies and specifying how, and to whom they are to be distributed, it was determined finally, on motion, that the Publishing Committee be instructed to publish, in pamphlet form, as many copies as in their judgment the finances of the Association would warrant, and that a copy be sent to each physician in the State, if possible.

Dr. S. R. Burroughs, chairman of committee appointed to consider and report upon the suggestions made by President Paine, reported as follows:

1. The Committee endorse the suggestions of the President in reference to ethics, and recommend a strict adherence to, and support of the same.

2. The suggestions as to a more practical and useful arrangement of the daily sittings of the Association are endorsed, and it is recommended that they be incorporated in the by-laws.

3. The amendment to article two of the constitution is endorsed and recommended.

4. In reference to suggestions on medical legislation, the report endorses and reaffirms every sentiment expressed, and asks that they be adopted and carried into execution as soon as possible.

5. The report is in accord with the suggestions as to suits for malpractice, and recommends that this Association place itself on record as favoring the sentiments expressed by the President.

6. The report approves all the suggestions as to the unjust revenue tax on surgical instruments and medicines, and the recommendations as to quarantine.

Dr. J. B. Robertson moved that the report be adopted, which was carried. He then moved that a committee of five be appointed to carry out the recommendations of the President and to incorporate them into the laws of the Association and report at the next meeting

Dr. Cummings seconded the motion, and it was carried, whereupon the chair reappointed the original committee.

Dr. Leake, chairman of the Special Auditing Committee, reported that the committee had examined and found correct the reports of the Secretary and Treasurer.

Dr. J. B. Robertson moved that the report be received and adopted. The motion was seconded and adopted.

The President then announced that section work would be resumed, and resigned his chair to Dr. J. C. Loggins, acting chairman of the

SECTION ON SURGERY.

Dr. Isaac E. Clark, of Schulenberg, read a paper on gunshot wounds in the abdomen. It was referred to the Publishing Committee without discussion.

Dr. C. F. Paine, of Comanche, read a paper on Fracture of the Superior Maxilla. It was referred without discussion.

Dr. A. W. Fly, of Galveston, exhibited an irrigating catheter, and explained its advantages.

Secretary Daniel moved that the other papers in this section be read by caption and referred. It was carried.

Dr. Sears moved that the section be closed and the next one be given a chance. This was seconded and lost.

Papers were then handed in to the Secretary by Dr. J. Braunnagel, of San Antonio, on comminuted fracture of skull and total loss of right anterior lobe of the brain; by Dr. J. M. Hays, of San Antonio, on suppurative inflammation of the liver; by Dr. G. A. Nelson, on gunshot wound in the forearm.

Dr. A. E. Spohn then read an exhaustive and entertaining paper on rabies and anti-rabic inoculation. After the referring of which paper to the usual committee the section was closed, and the

SECTION ON GYNECOLOGY

was opened, with Dr. B. E. Hadra, of Galveston, in the chair, and Dr. Pettus, of Galveston, Secretary. Dr. Hadra commenced the business of the section by reading a paper on the question: "How far abdominal surgery should be in the grasp of the general practitioners." The paper was referred.

Dr. Shropshire, of San Antonio, reported a case in which he used Alexander's operation. After reference, it was discussed by Drs. F. Herff, E. Cross, Hadra, E. J. Ward, and Wood.

Dr. Denton moved that in consideration of time the other papers in the section be read by caption and referred to the Publishing committee. The motion was seconded and adopted, and papers by Dr. Smiley, of Terrell; Dr. Lowry, of San Antonio; Dr. Lee, of Galveston, and Dr. F. H. Tucker, of San Augustine, were so referred.

The section was then closed, and the

SECTION ON OBSTETRICS

opened, Dr. Wilkes, of Waco, taking the chair, and Dr. Curtis, of Waco, being Secretary. The chairman read a paper entitled, "Gleanings from Obstetric Literature," which was duly referred.

Dr. C. F. Payne read a paper on the conservative treatment of lacerated perineum. Discussion followed, in which Dr. Wooten, of Austin, and Dr. Spohn, of Corpus Christi, took part.

Dr. T. J. Bell of Tyler read a paper on "Abortion and Premature Labor," which was discussed, and on motion was received and referred to the Publishing Committee.

On motion the section adjourned in order to go into general session to receive the report of the Nominating Committee.

The Nominating Committee reported as follows:

For President, Dr. R. M. Swearingen, of Austin.

First Vice President, Dr. A. Sims, McKinney.

Second Vice President, Dr. B. F. Kingsley, San Antonio.

Third Vice President, Dr. I. E. Clark, Schulenburg.

Chairman Section on Practice of Medicine, Materia Medica and Pathology, Dr. R. M. Ray, Whitewright.

Chairman Section on Obstetrics and diseases of children, Dr. S. D. Thruston, Dallas.

Chairman Section on Surgery and Anatomy. Dr. W. L. York, Decatur.

Chairman Section on Medical Jurisprudence, Chemistry and Psychology, Dr. A. D. Burroughs, Lovelady.

Chairman Section on State Medicine and Public Hygiene, Dr. E. L. Ward, Waxahachie.

Chairman Section on Gynecology, Dr. H. K. Leake.

Chairman Section on Ophthalmology and Otology, Dr. G. P. Hall, Galveston.

Chairman Section on Dermatology, Dr. S. H. Weatherford, Georgetown.

Chairman Section on Electro-Therapeutics, Dr. A. F. Sampson, Galveston.

The Committee on Legislation has been continued by the Association.

On Publishing Committee, Dr. Bennett, of Austin, in place of Dr. Swearingen.

Delegates to American Medical Association: O. Eastland, W. T. Jones, A. W. Pope, J. VanGaskin, B. W. Bristow, I. E. Clark, J. R. Briggs, F. L. Ford, F. E. Yoakum, T. J. Wagley, J. H. Sears and J. W. Garnett.

New members of the Judicial Committee: Drs. F. S. White, E. M. Rabb, J. V, Spring and J. T. Wagley.

The next place of meeting will be, on the fourth Tuesday in April, 1890, at Fort Worth.

Chairman of Committee of arrangement, Dr. W. P. Burts.

The report was received and adopted, after which the Secretary announced that he had been authorized to extend an invitation to the Association to visit the City Hospital.

On motion an adjournment took place until 3 o'clock.

AFTERNOON SESSION, THURSDAY APRIL 25.

At 3 P. M. the Association reconvened and President Paine announced that the

INSTALLATION OF THE NEWLY ELECTED OFFICERS, would be the first business of the evening. He appointed as a committee to escort them to the platform, Drs. J. H. Sears, S. R. Burroughs and H. C. Ghent.

PRESIDENT ELECT SWEARINGEN

on being introduced, said:

*Ladies and Gentlemen of the State Medical Association:*

"There are times and occasions when I find it extremely difficult for me to express myself as I would, and I am afraid that this is one of those occasions. I keenly appreciate the honor you confer upon my city, and I am painfully conscious of the responsibilities that that honor imposes upon me. For twenty years I have been an humble worker in the ranks of the State Medical Association. and in all those years I have thought of nothing—when subjects were brought up for consideration, I had but one aim, one object; to do what I thought was best for the State Medical Association. I never thought of sections. I never once thought of either North, South, East or West Texas; the one thought was always prominent in my mind, that we had but one Texas, and but one Texas State Medical Association. [Applause.] I wish we might forever eliminate from the counsels of this body of men all such thoughts as sections, and relegate them to the only place where it properly belongs, to the political arena. There it could have its influence in shaping the destinies of men, but not in the great field of seience; in this great work of humanity where we are all co-laborers, there can be no sections, there are no boundaries, no latitudes or longitudes. Science is as boundless as thought, and as eternal as the soul. Science has been defined by some good thinker, "an aggregation of knowledge simplified, and arranged so that one man can utilize it."

"We are all gatherers of knowledge; we are all workers in the field of science; the man from the country, from the town, the hamlet, the city; and we can all gather up our little facts and bring them together, and then these officers can arrange them in book form, and in book form they will be a science. We are completing and making science all the time as we push on with this work. We have a great work before us. This state of Texas might be allegorically called a garden for the production of the plant "*Scientia*," and this association is but the central point where we all come together and exhibit our fruits and flowers.

"Then let us still continue the work together, and I do earnestly trust that you will cooperate with the officers and help us to push forward to a splendid success the work that has been so well carried on in the past. I promise you now that under all circumstances and occasions I will unalterably follow—. At this point the speaker paused and said: "I beg pardon; I wanted to say something graceful of the gentleman who has just left the stage.

I have known him for many long years, since I was a boy, and from that time until now he has been my model and admiration. I will be as true as he has been and as earnest in upholding the honor and the dignity of our association.''

This brief speech was greeted with loud applause, which only ceased when Dr. J. F. Y. Paine came forward and made a graceful response, saying that in taking leave he thought it was his duty to express his sincere thanks for the forbearance they had exercised on all occasions for his short-comings as their presiding officer, and for the decorum and courtesy that had characterized their behavior on all occasions. In retiring he would carry with him all the affection and interest he had always had for the association, and he could assure them that whatever ability he possessed should be asserted in the promotion of the association's welfare.

The ontgoing president's speech was well received, and at the conclusion of it Dr. Ghent introduced Dr. Sims as the next vice-president of the grand old Texas State Medical Association. Dr. Sims made a brief and appropriate speech of thanks that was much applauded, then, owing to the absence of the other vice-presidents, the regular order of business was again taken up, the

SECTION OF OBSTETRICS

being reopened. Dr. R. G. Williams read a paper on two cases of puerperal convulsions which was freely discussed by Drs. L. J. Graham, Ghent, R. H. L. Bibb, Taylor, Fisher, Cummings, Fontaine, Paine and Sears, after which the section was closed.

THE SECTION ON STATE MEDICINE

was then opened, Dr. Leake acting chairman, and Dr. Ward Secretary.

A very interesting paper was read by Dr. Carhart, on Tyrotoxicon and Pepto-toxine, which was dicussed by Drs. Howard, Fontaine, C. F. Paine, Ghent, Leak, West and Coleman.

Dr. M. A. Taylor, of Austin, then read a paper on the Climate of West Texas, which was referred as usual, and the chairman then closed the section.

The business of the convention was then proceeded with, by the chair announcing that the two absent vice presidents, Drs. Clark and Kingsley, had come into the hall. He appointed Dr. Ward and Dr. Taylor to escort them to the stage. This being done, the two gentlemen were introduced, and both tersely expressed appreciation of the honor conferred on them by the Association, and gave assurances that their every effort would be bent to fill their positions acceptably.

After the conclusion of the applause consequent on the short speeches of the newly elected vice presidents, the President read a telegram from the Citizens' Committee of Corpus Christi, extending a welcome to the Association and stating that ample provision had been made for their reception and entertainment. Pres. Swearingen then said that a motion to adjourn until 9:30 next mornlng would be in order, whereupon his suggestion was carried out, and the Convention adjourned.*

FOURTH DAY, FRIDAY, APRIL 26.—MORNING SESSION.

The Convention was called to order at 9:30 A. M., by President Swearingen. Minutes of the previous day read and approved.

Dr. R. T. Knox nominated for honorary membership, Dr. Jos. T. Noyes, of Michigan and Dr. J. Harvey Reed, of Ohio. Referred to Judicial Council, which in order, reported favorably upon their names and both gentlemen were made honorary members of the Association. Dr. Noyes being present returned his cordial acknowledgements for the honor. The

SECTION ON OPHTHALMOLOGY

was called, Dr. R. H. Chilton Chairman. Dr. Chilton read his address, "Anæsthetics in Eye Troubles," which, on motion, was received and referred to Publishing Committee, as usual. There being no papers in this Section it was closed and the

SECTION ON ELECTRO-THERAPEUTICS

was called. The Chairman, Dr. W. M. Powell, of Albany, being

*Thursday night the President's address was delivered at the Opera-house, and a grand banquet was given at the Menger Hotel. Friday evening an excursion to Corpus Christi took place.

absent, and there being but two papers presented—they were, on motion read by caption and referred to Publishing Committee, to-wit: "Electricity in Obstetrics," and "Electro-Anæsthesia," both by Dr. F. T. Paine, of Comanche.

The hour having arrived for consideration of the report of the Committee on Constitution and By-Laws, which report was made special order for 10 A. M. to-day, Dr. Burroughs, acting Chairman, suggested the following as amendments to the Constitution and By-Laws:

1. That Article II of the Constitution of Texas State Medical Association be so amended as to read as follows:

"The objects of this Association shall be to organize the regular medical profession of the State in the most efficient manner possible; to encourage a high standard of professional qualification and ethics; to promote professional brotherhood; to labor for the advancement of State Medicine, i. e. of public hygiene; of medical education; of medical jurisprudence and public institutions for the sick and the infirm."

2. That Article III of said Constitution be so amended as to read as follows:

I. Every regularly educated man within the limits of this State, who is a graduate of a regular Medical College in good standing, and who adopts and conforms to the code of ethics of the American Medical Association, shall be eligible to membership in this body, provided they are members in good standing of their respective county or district medical societies.

II. In all cases where there exists no county medical organization, (and the qualifications for membership are perfect in every other respect) this fact shall not be a bar to membership.

We respectfully recommend that the last paragraph of that section of the Constitution and By-Laws which defines the powers and duties of the Judicial Council be so amended as to read as follows.

"All questions of a personal character, including complaints and protests, and all questions on credentials shall be referred at once to the Judicial Council and without discussion, whose decision shall be absolute and final on all questions of unscientific character of whatever nature, provided an appeal is not taken to the Judicial Council of the American Medical Association, in which case it shall be the duty of the General Secretary to furnish the Secretary of the higher Council with a complete and authenticated transcript of the proceedings of the lower Council.

SEC. 2. It shall be the duty of the Judicial Council, on all questions involving a penalty, after having arrived at a decision in any given case, as to the guilt or innocence of any member against whom charges of whatsoever nature may have been preferred, to assess the grade of punishment on the one hand, or declare an acquittal on the other, and report such action to the Association at its earliest convenience for its information; and, it is hereby made the duty of the General Secretary to record said report as part of the minutes of this Association.

SEC. 3. All questions based upon real or supposed professional or personal grievance must be adjudicated by the county or district societies of which the parties may be members, and it is hereby declared that all such questions are beyond the jurisdiction of the Judicial Council, provided no appeal from the lower council appears.

SEC. 3. In all questions of a personal or professional character arising between parties, either or both of whom reside in a county or counties where no recognized medical society exists, such cause shall fall under the jurisdiction of the nearest county or district medical association."

On motion the report of the committee was adopted; and Dr. Loggins moved that the above be incorporated in the Transactions as amendments to the Laws of the Association. Carried.

President Swearingen called Dr. Loggins to the chair and introduced the following preamble and resolutions :

"Whereas, The Railway Surgeons of America are called to meet in convention next month in the city of St. Louis, to confer as to ways and means, for perfecting the Hospital services of all systems, The Texas State Medical Association, by resolution, respectfully submits to them the following suggestions :

1st. A schedule of charges that requires a physician to charge a railway corporation, from fifty to one hundred per cent. less than he would charge his neighbor and friend for the same kind of service, insults our sense of justice, and violates the spirit of our ethics.

2. Any proposition to have the services of a local surgeon detailed in a report to his chief so as to enable said chief to estimate and be sole arbiter of the compensation to allow for said services, is degrading alike to our profession and to our manhood.

3. When injuries occur, by accident or otherwise, the local surgeon in charge of the case should always be consulted as to the physical ability of the injured to bear transportation to hospitals. An economy that rushes men on trains without the consent of the attending physician, is a discourtesy to him and sometimes fatal to the injured.

These suggestions are offered in no spirit of unkindness to a hospital system that certainly broadens the field of philanthropic usefulness, but the work should be done without bringing discredit upon men whose lives are devoted to the same noble service.

On motion of Dr. Taylor, the above resolution was adopted by a rising vote.

Dr. C. F. Paine offered a resolution suggesting to committees of arrangements in future to dispense with the banquet as a feature of the entertainment given the association. Adopted.

The Nominating Committee having neglected to appoint a Committee on Necrology, the chair appointed Drs. B. W. Bristow and Asa W. Pope.

Dr. T. J. Bennett offered the following resolution, which on motion was adopted:

"Resolved, That the President and Secretary of this Association be instructed to make application, without delay, to the Hon. Secretary of State for a charter incorporating the Texas State Medical Association."

Dr. J. B. Robertson announced the death of Dr. J. M. Ross, late of Brenham, a member of this Association, and paid a high tribute to his worth and ability as a physician and a citizen. The *data* in the life of Dr. Ross handed Dr. Robertson was, on motion, referred to the Committee on Necrology, and Dr. Robertson was requested to incorporate his eulogy in the report of said committee at next meeting.

[The usual resolutions of thanks to the citizens, the Press, the Ladies, the committee of arrangements, those who had extended invitation, etc., was adopted, also one to Mrs. Ford for a cake, and to the Y. M. C. A. for courtesies.—Ed.]

Dr. Evans offered the following resolution :

Resolved, That inasmuch as the Missouri Pacific Railroad Company extended to delegates to this convention a reduction of one-sixth from the usual fare—the Texas State Medical Association hereby return to them one-sixth of a vote of thanks. Adopted.

Dr. H. C. Ghent then delivered an address upon the subject of the Alamo monument, and said that all Texans should take a pride in subscribing to the noble work.

Dr. J. B. Robertson then introduced the following appropriate resolution, which, on motion, was adopted and ordered spread upon the minutes:

Resolved, That the effort to erect a monument to the heroes of the Alamo meets our hearty approval, and we pledge ourselves to do all in our power to promote the laudable undertaking.

Dr. A. N. Denton offered the following, which was adopted with an amendment making it a special committee :

Resolved, That it is the sense of this Association that the period in the history of Texas has been reached when the greatly increased population and rapidly growing commerce, demands for the protection and conservation of the public health an organized effort by the profession of the State, for the establishment of such sanitary laws as may be required to suit our changed condition.

Resolved, That the President of this Association is authorized and requested to appoint a committee of five members from this body who shall make an earnest effort to impress upon the members of the State legislature the importance of the establishment of a State Board of Health, and, if possible, secure an act for that purpose.

[The President has not yet made the appointment.—Ed.]

The Judicial Council reported favorably on the application for membership of the following physicians:

[The list is omitted here for want of room ; there were fifty-nine active and two honorary members elected.—Ed.]

On motion the convention adjourned to meet in Fort Worth on the fourth Tuesday in April, 1890.

F. E. Daniel, Secretary.

---

South Kansas Medical Society will meet at Wichita, Kansas, May 14, inst. Dr. W. K. Harris, Mulvane, Kansas, Secretary.

---

The Kansas State Medical Society met May 7, 8 and 9, inst., in Topeka, Kansas. Dr. J. E. Minney, Topeka, Secretary.

